# Validating and Improving Interrill Erosion Equations

**DOI:** 10.1371/journal.pone.0088275

**Published:** 2014-02-06

**Authors:** Feng-Bao Zhang, Zhan-Li Wang, Ming-Yi Yang

**Affiliations:** 1 State Key Laboratory of Soil Erosion and Dryland Farming on the Loess Plateau, Institute of Soil and Water Conservation, Northwest A&F University, Yangling, P. R. China; 2 Institute of Soil and Water Conservation, Chinese Academy of Science and Ministry of Water Resources, Yangling, P. R. China; University of Oxford, United Kingdom

## Abstract

Existing interrill erosion equations based on mini-plot experiments have largely ignored the effects of slope length and plot size on interrill erosion rate. This paper describes a series of simulated rainfall experiments which were conducted according to a randomized factorial design for five slope lengths (0.4, 0.8, 1.2, 1.6, and 2 m) at a width of 0.4 m, five slope gradients (17%, 27%, 36%, 47%, and 58%), and five rainfall intensities (48, 62.4, 102, 149, and 170 mm h^−1^) to perform a systematic validation of existing interrill erosion equations based on mini-plots. The results indicated that the existing interrill erosion equations do not adequately describe the relationships between interrill erosion rate and its influencing factors with increasing slope length and rainfall intensity. Univariate analysis of variance showed that runoff rate, rainfall intensity, slope gradient, and slope length had significant effects on interrill erosion rate and that their interactions were significant at p = 0.01. An improved interrill erosion equation was constructed by analyzing the relationships of sediment concentration with rainfall intensity, slope length, and slope gradient. In the improved interrill erosion equation, the runoff rate and slope factor are the same as in the interrill erosion equation in the Water Erosion Prediction Project (WEPP), with the weight of rainfall intensity adjusted by an exponent of 0.22 and a slope length term added with an exponent of −0.25. Using experimental data from WEPP cropland soil field interrill erodibility experiments, it has been shown that the improved interrill erosion equation describes the relationship between interrill erosion rate and runoff rate, rainfall intensity, slope gradient, and slope length reasonably well and better than existing interrill erosion equations.

## Introduction

Soil erosion on a hill slope consists of two major components: interrill and rill erosion [1]. Interrill erosion is the detachment and transport of soil material from the surface of the soil matrix by raindrop impact and overland flow [2]. More recent process-based models, e.g., WEPP [3], EUROSEM [4], LISEM [5], and PSEM-2D [6], make an explicit distinction between rill and interrill erosion processes and estimate interrill erosion rate using a function of various factors, including interrill erodibility parameter which expresses the soil's resistance to interrill erosion within interrill areas, rainfall erosivity, slope characteristics, hydraulic factors, vegetation, and land use [4]–[10].

It is difficult to describe and quantify interrill and rill erosion processes simultaneously during a rainfall event, especially to measure the rate of interrill erosion because it is less visible. The mini-plot, which eliminates the effect of rill erosion, has been an important means to study interrill erosion and to establish interrill erosion equations. The effects of rainfall intensity, slope length, slope gradient, runoff rate, and soil type on interrill erosion rate have been described. Power functions were used to describe the effect of rainfall intensity on interrill erosion. The rainfall intensity exponent varied from 1.6 to 2.3 [11] and from 1.36 to 2.54 [12]. The exponent decreased as the clay content of the soil increased [11]. Therefore, interrill erosion rate has been shown to be approximately proportional to the square of the rainfall intensity for a given mini-plot if runoff is not considered [12]–[15]. To describe the effect of slope gradient on interrill erosion, several formulas of slope factors have been proposed [16]–[19]. The role of runoff was initially considered as sediment transport for interrill erosion [20] and was not included in interrill erosion equations [3], but some researchers found that model predictability was improved when a separate runoff parameter was included in interill erosion equations [21], [22]. In general, interrill erosion rate increased gradually with increasing runoff. For the effect of slope length on interrill erosion, there have been inconsistent results, which will be described detailedly in the following discussion.

According to the relationships between interrill erosion rate and its influencing factors, interrill erosion equations have been proposed on the basis of results from mini-plot experiments. In the WEPP model, the following equation was first used to estimate interrill erosion rates [3]:
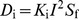
(1)where *D*_i_ is interrill erosion rate (kg m^−2^ s^−1^), *K*_i_ is interrill erodibility, *I* is rainfall intensity (m s^−1^), and *S*_f_ is slope factor that can be calculated using the following equation [16]:
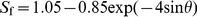
(2)where *θ* is the slope gradient (°).

As described above, previous studies have shown that model predictability was improved when a separate runoff parameter was included in interrill erosion equations [21], [22]. Therefore, Eq. (1) was modified by adding the factor of overland flow [22] and was then used in the WEPP model in 1995 as the following formula [23]:
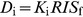
(3)where *R* is runoff rate (m s^−1^). In addition, to describe interrill erosion processes more accurately and to develop a simple lumped parameter model, the following equation was proposed by analyzing simulated rainfall data [19]:
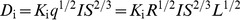
(4)where *q* is flow discharge per unit width (m^2^ s^−1^) and *S* is slope inclination (m m^−1^). This equation thought that the rainfall intensity term, *I*, represented the detachment of soil by raindrop impacts and the enhancement of the transport capacity of overland flow and that the term *q* ^1/2^ *S* ^2/3^ represented sediment transport by thin overland flow. Compared to Eq. (3), the effect of runoff on interrill erosion rate was weakened, and the positive effect of slope length on interrill erosion rate was added to Eq. (4).

Parameters for soil type (*K*_i_), slope gradient, rainfall intensity, and overland flow are included in Eqs. (1), (3), and (4). The parameter for slope length is included only in Eq. (4). However, inconsistent results have been reported for the effect of slope length on interrill erosion. Running the similar experiments which simulated slope length using inflow rates, some experiments indicated consistently greater interrill erosion from increased overland flow [24], but other experiments showed that greater discharge rates resulted in decreased soil loss [25], [26]. The authors thought that interrill erosion at a particular downslope distance is dictated by the soil detachment or sediment transport capacity characteristics existing at that particular location [25], [26]. The effects of row-sideslope length within a range of 0.15 m to 0.6 m on sideslope erosion for four soils at several rain intensities demonstrated that slope length affected erosion very little until the soils began to rill, but had a major effect once rilling occurred [27]. However, the inconsistent viewpoint, which was that the effect of slope length on interrill erosion might have been due to a change from erosion which is dominated by raindrop-induced flow transport (RIFT) to erosion which is dominated by raindrop detachment-flow transport (RD-FT) except for the effect of slope length on rilling, was concluded by using the same data [28]. Other experiments indicated that soil losses were significantly correlated with rainfall intensity (*r* = 0.89; P<0.001) and slope length (*r* = 0.43; P<0.001), and suggested that careless scale transfer of erosion data may lead to erroneous conclusions [29]. In addition, some researchers have attributed the effect of slope length on interrill erosion to rainfall intensity and soil properties [27], [30] and have assumed that slope length has little or no effect on interrill erosion per unit of area [31], [32]. In short, although numerous studies were reported, the effect of slope length on interrill erosion has not been clearly established.

In addition, Eqs. (1), (3), and (4) were developed for smaller plot sizes (less than 0.5 m^2^), shorter slope lengths (less than 0.8 m), and small slope gradients, and the different researchers who developed these equations used different plot sizes and slope lengths [3], [19], [22], [27]. They suggested that the applicability of their model to longer slopes required further testing [19]. To date, no systematic experiments based on mini-plot experiments have been conducted to validate and compare the existing interrill erosion equations for different slope lengths or plot sizes and a wider range of slope gradients and rainfall intensities. The objective of the present study is to validate the existing interrill erosion equations under different controlled experimental conditions and to describe interrill erosion rate accurately by adding or adjusting factors in the interrill erosion equations.

## Materials and Methods

Erosion tests were conducted at the State Key Laboratory of Soil Erosion and Dryland Farming on the Loess Plateau in Yangling, China. Artificial rainfall was produced using a rain-making machine with an effective rainfall area of 2 m×3 m at a height of 8.67 m, which produced a simulated rainstorm at a controllable intensity with distribution uniformity greater than 85% and a median raindrop diameter of 2.2 mm, comparable to natural rainfall.

The soil used in the tests was cultivated Huangmian soil (Calcaric Cambisols, FAO) from Ansai County in Shaanxi Province, located in the northern part of the Loess Plateau. The cultivated land sampled in this study is publicly owned and managed by the Ansai Research Station of Soil and Water Conservation, Chinese Academy of Science. No specific permits were required for the field studies described. The soil contained 38.72% sand, 45.59% silt, 15.69% clay, and 0.53% organic matter. The soil was collected from the top 0.25 m layer in cultivated land, air-dried, crushed to pass through a 4-mm sieve, and thoroughly mixed. The soil moisture content was held constant at 14% for all experiments.

Perforated metal flumes with different lengths (0.4, 0.8, 1.2, 1.6, and 2 m), 0.4 m in width and 0.25 m in depth, were manufactured for use in the tests. The slope gradient of each flume could easily be adjusted from 0% to 84%. A trough was placed at the lower edge of the flume to collect runoff and sediment samples. The soil was packed uniformly into the flumes in four 5.5-cm layers to a total depth of 22 cm with a bulk density of 1.3 g cm^−3^. To reduce discontinuities between layers, the surface of each soil layer was gently scored. The surface of the top layer was smoothed to minimize microtopographic effects.

Two replications of all combinations of one soil type, five levels of slope length (0.4, 0.8, 1.2, 1.6, and 2 m), five levels of slope gradient (17%, 27%, 36%, 47%, and 58%), and five levels of rainfall intensity (48, 62.4, 102, 149, and 170 mm h^−1^) were tested using a randomized factorial design. A total of 250 runs were conducted. Replicate means were used in the data analyses.

Simulated rainfall was generally applied for 60 min for each rainfall event and was terminated if rill erosion occurred during the 60 min. Sediment and runoff samples were collected continuously throughout the rainfall event. Samples were collected at 1, 2, 3, 4, and 5 min within the first 15 minutes after runoff production and then every 5 min thereafter. Sediment and runoff were measured gravimetrically. In general, steady states of soil loss and runoff rate were reached within 30 min after runoff commenced. To minimize the effect of surface condition, steady-state values were used in the analysis [3], [19], [27]. Values identified as outliers were not included [3]. In total, 36 of 1740 experimental values and 22 of 740 steady-state values were rejected. Statistical analyses were performed using the SPSS PASW Statistics (Version 18.0) software.

## Results and Discussion

### Validation of existing interrill erosion equations

**Figure 1:**
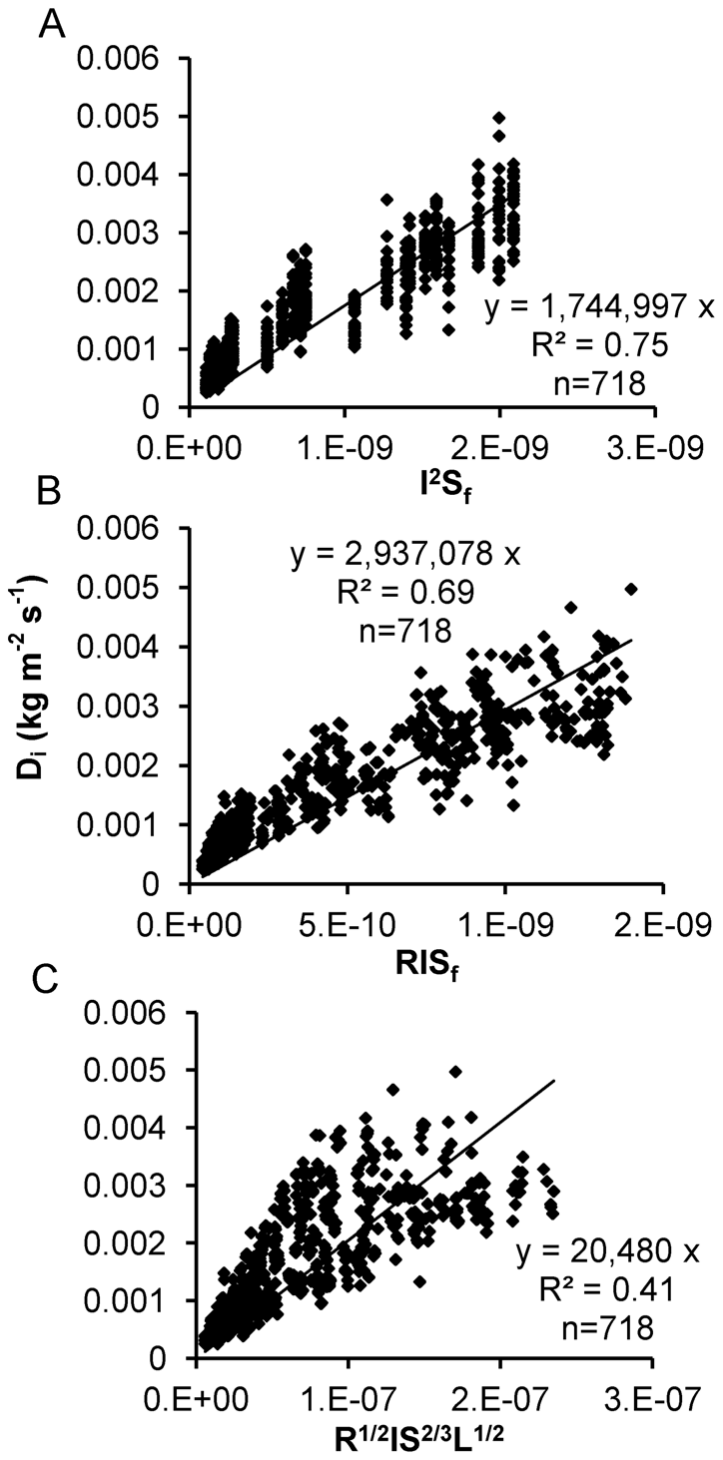
Linear regressions with zero intercept. Using linear regression with zero intercept between interrill erosion rate and the scaling factors I^2^S_f_ (A), RIS_f_ (B), and IR^1/2^S^2/3^L^1/2^ (C) to assess the previous interrill equations based on the mini plots.

Interrill erodibility based on mini-plot experiments for a given soil type was defined as a constant for each equation. This constant was determined from linear regression between the interrill erosion rate *D*_i_ and the scaling factors *I* ^2^ *S*_f_ in Eq. (1), *RIS*_f_ in Eq. (3), and *IR* ^1/2^ *S* ^2/3^ *L* ^1/2^ in Eq. (4) for all effective steady-state values. A zero intercept was used in the regression, and the slope of the regression line was assumed to be the interrill erodibility (*K*_i_). Figure 1 presents the results of linear regression with zero intercept for Eqs. (1), (3), and (4). Good linear regressions could not be gained for the three interrill erosion equations for all effective steady-state values, which indicated that in some cases the existing interrill erosion equations did not adequately describe the relationship between interrill erosion rate and its influencing factors.

**Figure 2:**
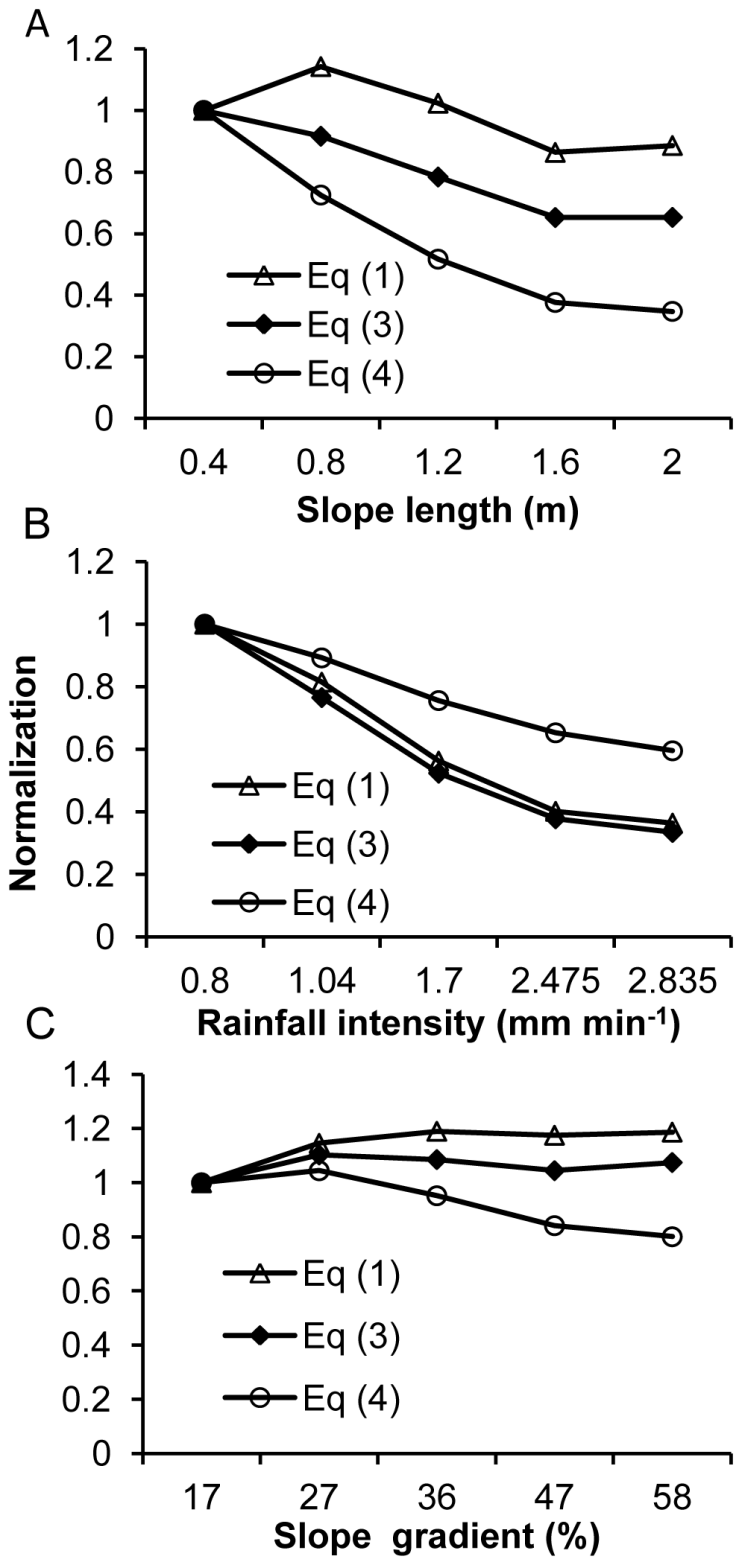
Normalized interrill erodibilities. Variations in interrill erodibility for the three equations for different slope lengths (A), rainfall intensities (B), and slope gradients (C), normalized to the lowest values of the independent factors.

To find further key factors influencing the equation's predictive power, interrill erodibility was also calculated using linear regression with a zero intercept under various controlled experimental conditions. Table 1 presents interrill erodibility (*K*_i_) and coefficient of determination (*R* ^2^) for different slope lengths for the three existing equations. When rainfall intensity and slope gradient were varied and slope length was increased from 0.4 m to 2 m, the interrill erodibility (*K*_i_) from Eq. (1) varied slightly, with a coefficient of variation (CV) of 11%, whereas that from Eq. (3) decreased by approximately 1.5 times, with a CV of 19%, and that from Eq. (4) varied widely and decreased by approximately 2.9 times, with a CV of 46% (Fig. 2a). These results indicated that slope length plays a key role in Eqs. (3) and (4) and should be included in these equations. Compared with Eq. (1), the wide variations in interrill erodibility for Eqs. (3) and (4) might imply interactions between runoff and slope length. The change in the coefficient of determination (*R* ^2^) corresponding to the change in slope length from 0.4 m to 2 m demonstrated that the three existing interrill equations could describe interrill erosion processes effectively for plots with shorter slope lengths, especially those experiments of approximately 0.4 m. This finding is consistent with existing interrill erosion equations established on the basis of data from mini-plots with shorter slope lengths in previous works [3], [12], [27].

**Table 1:** Interrill erodibilities (*K*_i_) and coefficients of determination (*R* ^2^) for Eqs (1), (3) and (4) for different slope lengths.

L (m)		Eq (1)	Eq (3)	Eq (4)
0.4	K_i_	1,773,995	3,734,418	43,430
	R^2^	0.90	0.90	0.96
0.8	K_i_	2,026,011	3,420,684	31,387
	R^2^	0.77	0.78	0.88
1.2	K_i_	1,815,288	2,927,228	22,287
	R^2^	0.79	0.80	0.84
1.6	K_i_	1,531,958	2,435,898	16,294
	R^2^	0.71	0.70	0.82
2.0	K_i_	1,571,555	2,437,679	15,029
	R^2^	0.64	0.64	0.77
Mean	K_i_	1,743,761	2,991,181	25,686
CV		11%	19%	46%

Table 2 presents the interrill erodibility (*K*_i_) values and coefficients of determination (*R* ^2^) obtained for different rainfall intensities using the three existing equations. Interrill erodibility (*K*_i_) for the three equations decreased as rainfall intensity increased from 0.8 to 2.835 mm min^−1^ (Fig. 2b). Interrill erodibility (*K*_i_) varied obviously for Eqs. (1) and (3), with a CV of approximately 45%, but varied relatively little for Eq. (4), with a CV of 18% because of the decrease in the weight of runoff rate in the equation. These results indicated that the effects of rainfall intensity or runoff rate are overestimated by the three existing interrill erosion equations. When the weight of runoff or rainfall intensity in the interrill erosion equations was decreased, the equations' predictive power was improved. Therefore, an adjustment to these weights should be considered.

**Table 2:** Interrill erodibilities (*K*_i_) and coefficients of determination (*R* ^2^) for Eqs. (1), (3), and (4) for different rainfall intensities.

I (mm min^−1^)		Eq (1)	Eq (3)	Eq (4)
0.8	K_i_	4,386,185	7,967,808	29,897
	R^2^	0.26	0.49	0^a^
1.04	K_i_	3,572,441	6,097,944	27,063
	R^2^	0.39	0.45	0
1.7	K_i_	2,465,436	4,171,479	23,615
	R^2^	0.4	0.37	0
2.475	K_i_	1,761,220	3,009,673	20,424
	R^2^	0.6	0.39	0
2.835	K_i_	1,596,803	2,662,866	19,345
	R^2^	0.46	0.17	0
Mean	K_i_	2,756,417	4,781,954	24,069
CV		43%	47%	18%

a *R^2^* was negative for the linear regression with zero intercept between interrill erosion rate and Iq^1/2^S^2/3^ in Eq (4); therefore, it was replaced by zero.

Table 3 presents the interrill erodibility (*K*_i_) values and coefficients of determination (*R* ^2^) obtained for different slope gradients for the three existing equations. Only for Eq. (4) did interrill erodibility decrease with an increase in slope gradient (Fig. 2c). In general, interrill erodibility from these three equations varied slightly with slope gradient. These results suggested that the effect of slope gradient on interrill erosion rate is almost completely expressed by the existing equations, but that use of the *S*_f_ term in Eqs. (1) and (3) yields better results than use of the *S* ^2/3^ term in Eq. (4). The lower coefficient of determination (*R* ^2^) values shown in Tables 2 and 3 also implied that slope length is the most important factor affecting interrill erosion processes and results in lower coefficients of determination (*R* ^2^).

**Table 3:** Interrill erodibilities (*K*_i_) and coefficients of determination (*R* ^2^) for Eqs. (1), (3), and (4) for different slope gradients.

S (%)		Eq (1)	Eq (3)	Eq (4)
17	K_i_	1,503,203	2,753,304	23,327
	R^2^	0.71	0.7	0.47
27	K_i_	1,722,260	3,036,752	23,789
	R^2^	0.74	0.68	0.44
36	K_i_	1,788,967	2,989,530	21,976
	R^2^	0.72	0.65	0.37
47	K_i_	1,766,816	2,875,945	19,512
	R^2^	0.71	0.64	0.34
58	K_i_	1,784,369	2,958,936	18,592
	R^2^	0.75	0.67	0.34
Mean	K_i_	1,713,123	2,922,893	21,439
CV		7%	4%	11%

In summary, the results from the analysis described above suggested that the three existing interrill erosion equations considered do not completely describe the relationship between interrill erosion rate and its influencing factors, especially slope length and rainfall intensity or runoff. In addition, univariate analysis of variance was conducted on the assumption that runoff rate was the covariant and that rainfall intensity, slope gradient, and slope length were the fixed factors. The results of this analysis showed that runoff rate, rainfall intensity, slope gradient, and slope length had significant effects on interrill erosion rate and that their interactions were significant at p<0.01 (Table 4). These results also implied that the effect of slope length on interrill erosion rate should be considered in the interrill erosion equations. Therefore, an interrill erosion equation is needed that includes a factor for slope length and weight adjustments for the other factors.

**Table 4:** Univariate Analysis of Variance for selected parameters with interrill erosion rate as a dependent variable using all effective steady-state values.

Source	Type III Sum of Squares	df	Mean Square	F Value	Sig.
Corrected Model	.001^a^	125	5.405E-06	115.591	.000
Intercept	2.797E-06	1	2.797E-06	59.824	.000
Runoff (R)	2.747E-07	1	2.747E-07	5.874	.016
Intensity (I)	3.518E-06	4	8.794E-07	18.808	.000
Slope length (L)	1.939E-05	4	4.847E-06	103.660	.000
Slope gradient (S)	3.017E-05	4	7.542E-06	161.305	.000
I * L	1.104E-05	16	6.901E-07	14.760	.000
I * S	1.507E-05	16	9.419E-07	20.144	.000
L * S	8.892E-06	16	5.558E-07	11.886	.000
I * L * S	8.576E-06	64	1.340E-07	2.866	.000
Error	2.768E-05	592	4.676E-08		
Total	.003	718			
Corrected Total	.001	717			

a R^2^ = 0.961 (Adjusted R^2^ = 0.952).

### Improved interrill erosion equation

According to Eqs. (1), (3), and (4) and the results of the analysis described above, the interrill erosion rate for a given soil type can be described by a function of the following general form:
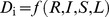
(5)


In this function, slope gradient (*S*), rainfall intensity (*I*), and slope length (*L*) are three independent variables that directly affect runoff rate (*R*) and directly or indirectly affect interrill erosion rate (*D*_i_). Runoff rate also affects interrill erosion rate. In fact, these factors are interdependent and interactive. Multivariate regression does obtain the best-fit expression, but does not reflect the physical interpretation of the interaction between interrill erosion rate and its influencing factors. It is difficult to eliminate the interdependence and interaction among these factors and to express accurately the relationship between interrill erosion rate and its influencing factors using multivariate regression. Therefore, before structuring the interrill erosion equation, it is necessary to analyze individually the relationships between interrill erosion rate and each influencing factor and to understand their physical interpretation under controlled conditions.

Sediment concentration (*C*) is a composite indicator whose variations reflect the dynamic variations of runoff and soil loss. Transport by interrill overland flow is the predominant process in interrill erosion [2], and the detachment process can be considered negligible [20]. Based on the theory of erosion by rainfall-disturbed flow [33], [34], sediment concentration was determined by soil factors, rainfall intensity, kinetic energy per unit quantity of rain, slope length, and slope gradient [28]. Because the kinetic energy of rain is an expression of rainfall intensity [35], the sediment concentration for a given soil type can also be expressed by a function of the following general form:
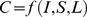
(6)


Compared with Eq. (5), there is one dependent parameter (*C*) in Eq. (6), and the effects of the interdependence between runoff rate and rainfall intensity, slope gradient, and slope length in Eq. (5) are not considered in Eq. (6), while interactions among rainfall intensity, slope gradient, and slope length may exist in Eq. (6).

Sediment concentration (*C*) can also be calculated by the following formula:
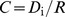
(7)


Therefore, if the relationships between sediment concentration and slope gradient, rainfall intensity, and slope length can be determined, the relationships between interrill erosion rate and runoff rate, slope gradient, rainfall intensity, and slope length can also be determined. The relationships between sediment concentrations and each influencing factor are quantified in the following discussion.

**Figure 3:**
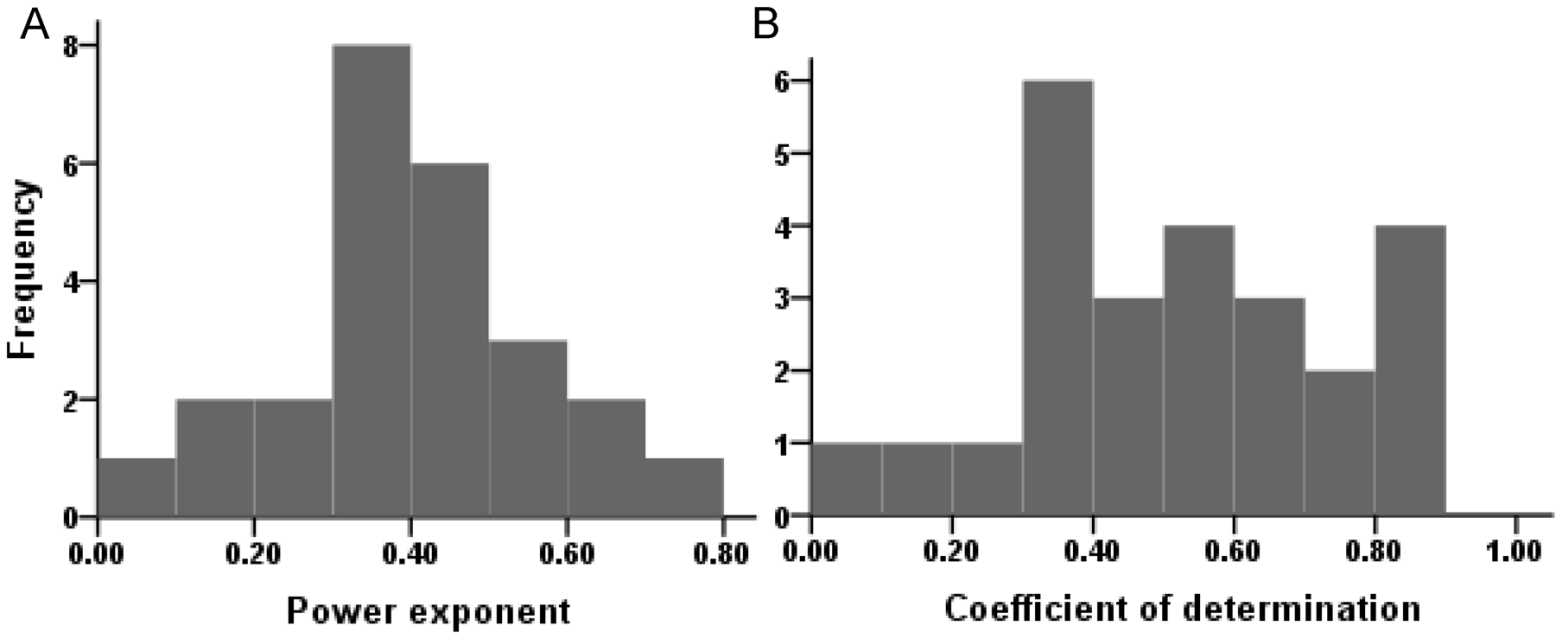
Regression parameters of power function. Power exponents (A) and coefficients of determination (B) for the relationship between sediment concentration and slope gradient for all tests.

For the same slope length and rainfall intensity, highly significant power-function relationships (p<0.05) between sediment concentration and slope gradient were found except for one combination test, although the power exponents were different for different combinations of slope length and rainfall intensity (Fig. 3). In general, the change in sediment concentration was less than linearly related to the change in slope gradient, with a mean power exponent of 0.4 and a CV of 0.41. The power exponents and the coefficients of determination (*R* ^2^) decreased with increasing slope length. This suggested that the effect of slope gradient on sediment concentration decreases with increasing slope length. The variability of the power exponent was less for higher rainfall intensities than for lower rainfall intensities.

**Figure 4:**
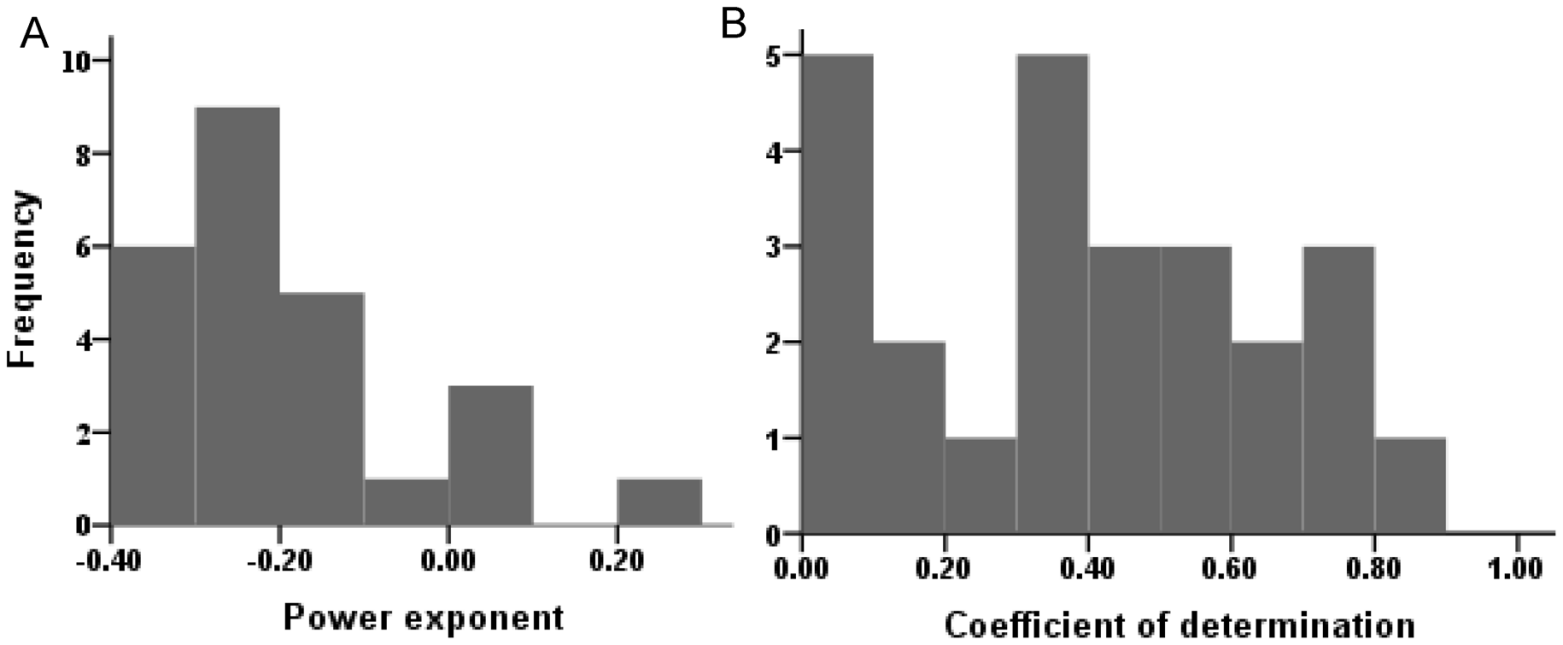
Regression parameters of power function. Power exponents (A) and coefficients of determination (B) for the relationship between sediment concentration and slope length for all tests.

For the same slope gradient and rainfall intensity, significant negative power-function relationships (p<0.05) between sediment concentration and slope length were detected in 19 out of 25 combination tests, and one had a negative power-function relationship (p>0.05). Figure 4 presents the frequencies of the power exponents and the coefficients of determination of the relationship between sediment concentration and slope length for all tests. For five combination tests with positive exponents, four tests were insignificant (p>0.05), and one had a significantly positive correlation (p<0.05). The positive power exponents between sediment concentration and slope length might have resulted from poor control of the experimental conditions or from other random factors. The mean power exponent of the negative correlation was −0.25, with a CV of 0.33. In general, the power exponents decreased as slope gradient and rainfall intensity increased. The negative correlation between sediment concentration and slope length might indicate that the role of raindrop detachment on detached soil particles from the soil surface weakens with increasing slope length and that transport-limited erosion processes transfer to detachment-limited erosion with increasing slope length.

**Figure 5:**
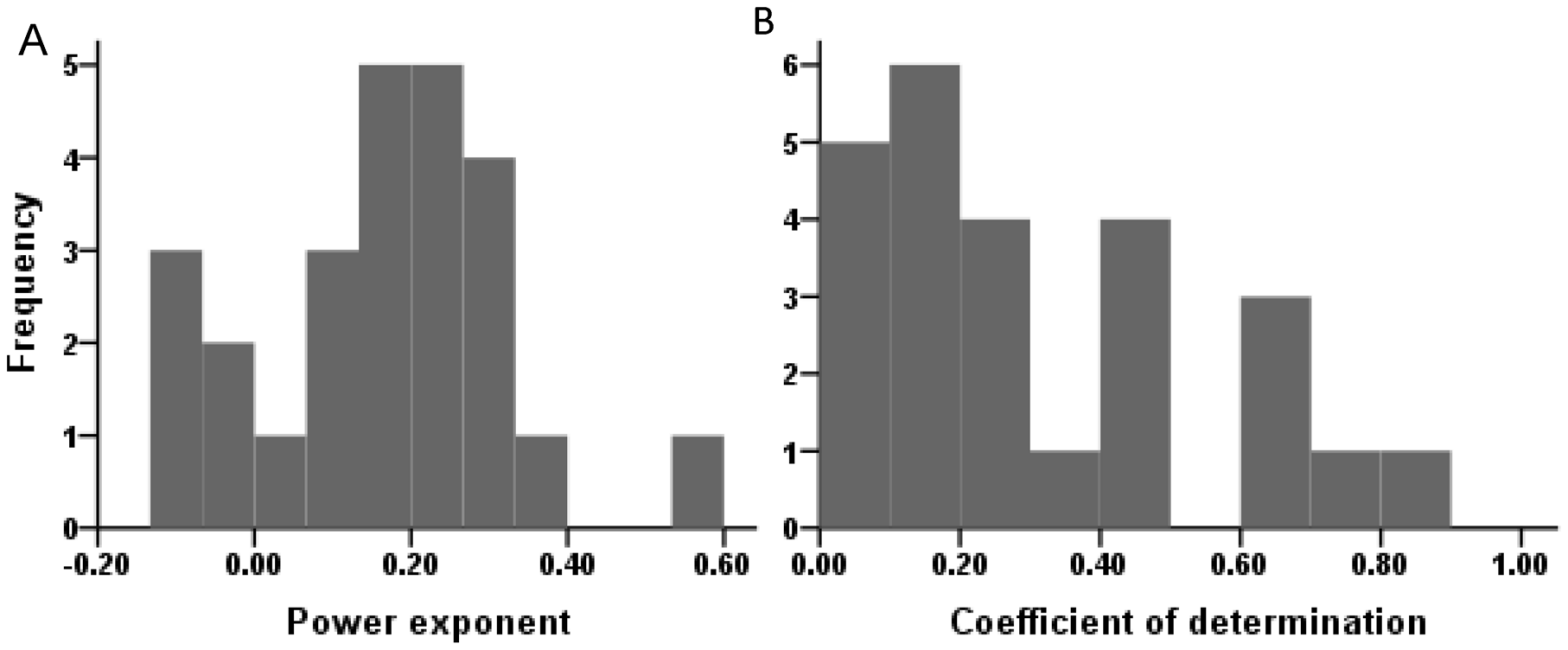
Regression parameters of power function. Power exponents (A) and coefficients of determination (B) for the relationship between sediment concentration and rainfall intensity for all tests.

A power-function relationship between sediment concentration and rainfall intensity was derived for controlled conditions of fixed slope length and gradient. Among the 25 combination tests, 18 were significant positive power-function relationships (p<0.05), 2 were insignificant positive power-function relationships (p>0.05), and 5 were insignificant negative power-function relationships (p>0.05). These results indicate that positive power-function relationships exist between sediment concentration and rainfall intensity, while for a few tests, an insignificant negative correlation was detected, and that the power exponents are different for different tests (Fig. 5). The mean power exponent of the positive correlations was 0.22, with a CV of 0.55. In general, the power exponents decreased as slope length increased, which indicated that the effect of rainfall intensity on sediment concentration weakens due to the increase of flow depth and the reduction in raindrop detachment with increasing slope length and plot size.

Based on the results of the analysis described above, power-function relationships were derived between sediment concentration (*C*) and slope gradient (*S*), rainfall intensity (*I*), and slope length (*L*) for all combination tests. The averaged power exponents were used as a basis for describing the relationship between C and I, S, and L in Eq. (6). As a result, Eq. (6) can be expressed as:
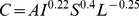
(8)


In Eq. (8), A is a constant for a given soil type and is equivalent to the interrill erodibility (Ki) of that soil type.

Combining Eqs. (7) and (8), the interrill erosion rate, *D*_i_ (kg m^−2^ s^−1^), can be expressed by the following equation:
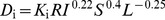
(9)


However, as the previous work noted [19], the exponents of rainfall intensity, slope gradient, and slope length can be slightly altered to account for interactions among soil type, gradient, slope length, and rainfall intensity.

**Figure 6:**
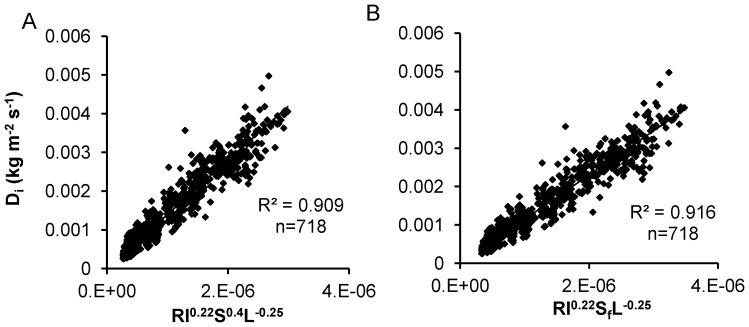
Linear regressions with zero intercept. Linear regression with zero intercept between the interrill erosion rate and the scaling factors R I^0.22^S^0.4^L^−0.25^ (A) and R I^0.22^ S_f_ L^−0.25^ (B) to assess the slope factor of S^0.4^ and S_f_.

Figure 6 shows linear regressions with zero intercept between the interrill erosion rate (*D*_i_) and the scaling factor *RI* ^0.22^ *S* ^0.4^ *L* ^−0.25^ and the scaling factor *RI* ^0.22^ *S*_f_ *L* ^−0.25^. Although the coefficient of determination (*R* ^2^) values using *S* ^0.4^ and *S*_f_ were not significantly different, using *S*_f_ to describe the effect of gradient on interrill erosion yielded slightly better results than using *S* ^0.4^. Moreover, using *S*_f_ was consistent with results obtained by other researchers [16]. Equation (9) could therefore be transformed as follows:
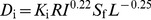
(10)


To assess the performance of Eq. (10), table 5 presents the linear regression slopes (*K*_i_) and coefficients of determination (*R* ^2^) obtained from linear regressions with zero intercept between the interrill erosion rate (*D*_i_) and the scaling factor *RI* ^0.22^ *S*_f_ *L* ^−0.25^ for different experimental conditions. The higher coefficients of determination (*R* ^2^) and the lower CVs of interrill erodibility (*K*_i_) indicated that Eq. (10) is better than previously developed equations for interrill erosion and adequately predicts interrill erosion for a wide range of rainfall intensities, slope gradients, and slope lengths.

**Table 5:** Slopes of the linear regression (*K*_i_) and coefficients of determination (*R* ^2^) from Eq (10) for different experimental conditions.

L (m)		Eq (10)	I (mm min^−1^)		Eq (10)	S (%)		Eq (10)
0.4	K_i_	1,132	0.8	K_i_	1,271	17	K_i_	1,082
	R^2^	0.95		R^2^	0.5		R^2^	0.86
0.8	K_i_	1,234	1.04	K_i_	1,190	27	K_i_	1,197
	R^2^	0.95		R^2^	0.49		R^2^	0.89
1.2	K_i_	1,163	1.7	K_i_	1,192	36	K_i_	1,182
	R^2^	0.93		R^2^	0.57		R^2^	0.92
1.6	K_i_	1,050	2.475	K_i_	1,148	47	K_i_	1,134
	R^2^	0.90		R^2^	0.72		R^2^	0.9
2.0	K_i_	1,153	2.835	K_i_	1,134	58	K_i_	1,140
	R^2^	0.86		R^2^	0.67		R^2^	0.94
Mean	K_i_	1,146	Mean	K_i_	1,187	Mean	K_i_	1,147
CV		5.7%	CV		4.5%	CV		3.9%

**Figure 7:**
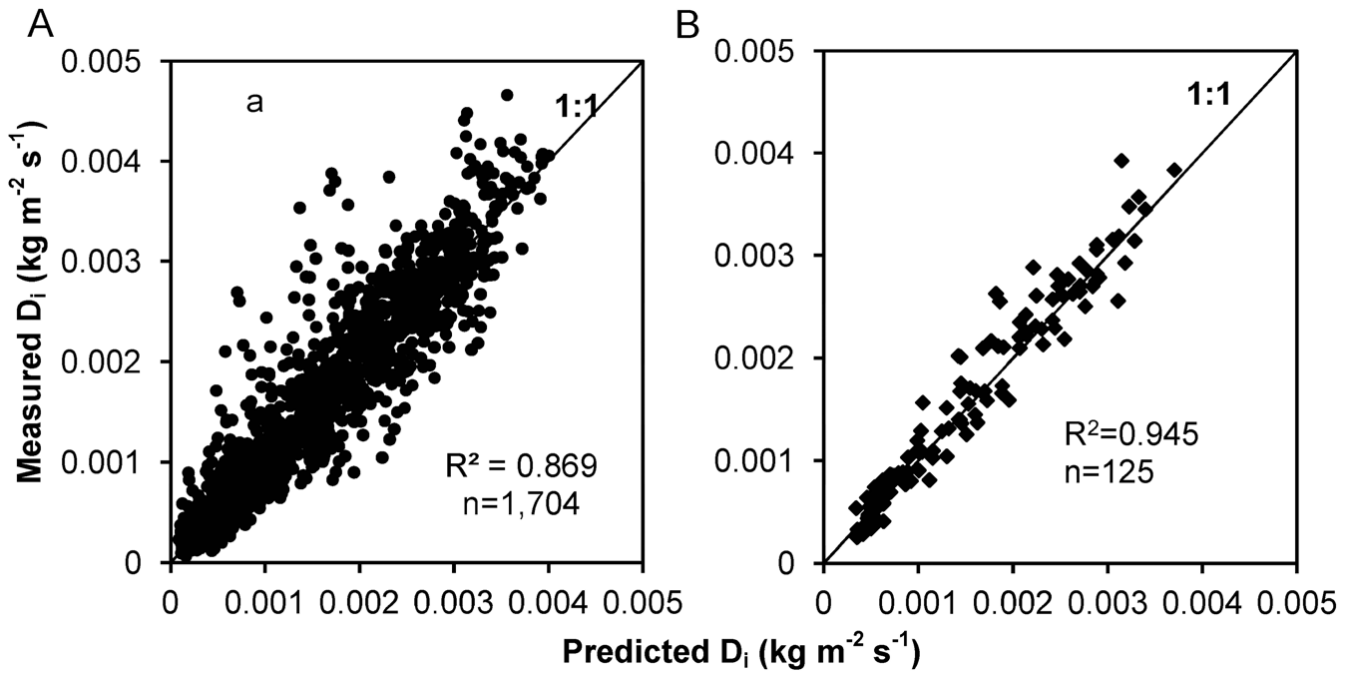
Measured vs. predicted interrill erosion rates. Measured and predicted interrill erosion rates for Eq. (10) for the erosion processes during a rainfall event (A) and the average of each rainfall event (B).

As stated previously, Eq. (10) was developed for steady-state overland flow and interrill erosion rate. Equation (10) must be validated for non-steady-state conditions. Equation (10) was therefore used to predict interrill erosion rates during a rainfall event and the averaged interrill erosion rate of each rainfall event for all rainfall events in this study. The measured and predicted interrill erosion rates are presented in Fig. 7. Correlation coefficients of 0.932 and 0.972 between the measured and predicted interrill erosion rates were obtained, and Nash-Sutcliffe coefficients of 0.865 and 0.942 were obtained for the interrill erosion rates of erosion processes and the average of each rainfall event respectively. These results suggested that Eq. (10) could predict interrill erosion rates reasonably well, despite a few predicted values that were considerably different from the corresponding measured values.

### Validation of the improved interrill erosion equation

To evaluate further the effectiveness of the improved interrill erosion equation, experimental data from WEPP cropland soil field interrill erodibility experiments were used for validation. These experiments were part of the USDA-ARS WEPP project, which included thirty-three soils from all areas of the United States in ridge plots. Flat plots were excluded from the validation because their slope gradients were much less than the lowest slope gradient in these experiments and some soil types were not represented in flat plots. The detailed information about the experimental materials and procedures were described [3]. Based on their data analysis techniques, the means of the last four erosion rates and flow discharges were selected as the erosion rate and flow discharge for a given plot in this validation. If there was an outlier value within the last four, then it was not considered, and the four most consistent of the last five values were used [3]. Although the fixed slope length was an obstacle to validating the improved interrill erosion equation, these experimental data could be used to judge the quality of different interrill erosion equations.

**Figure 8:**
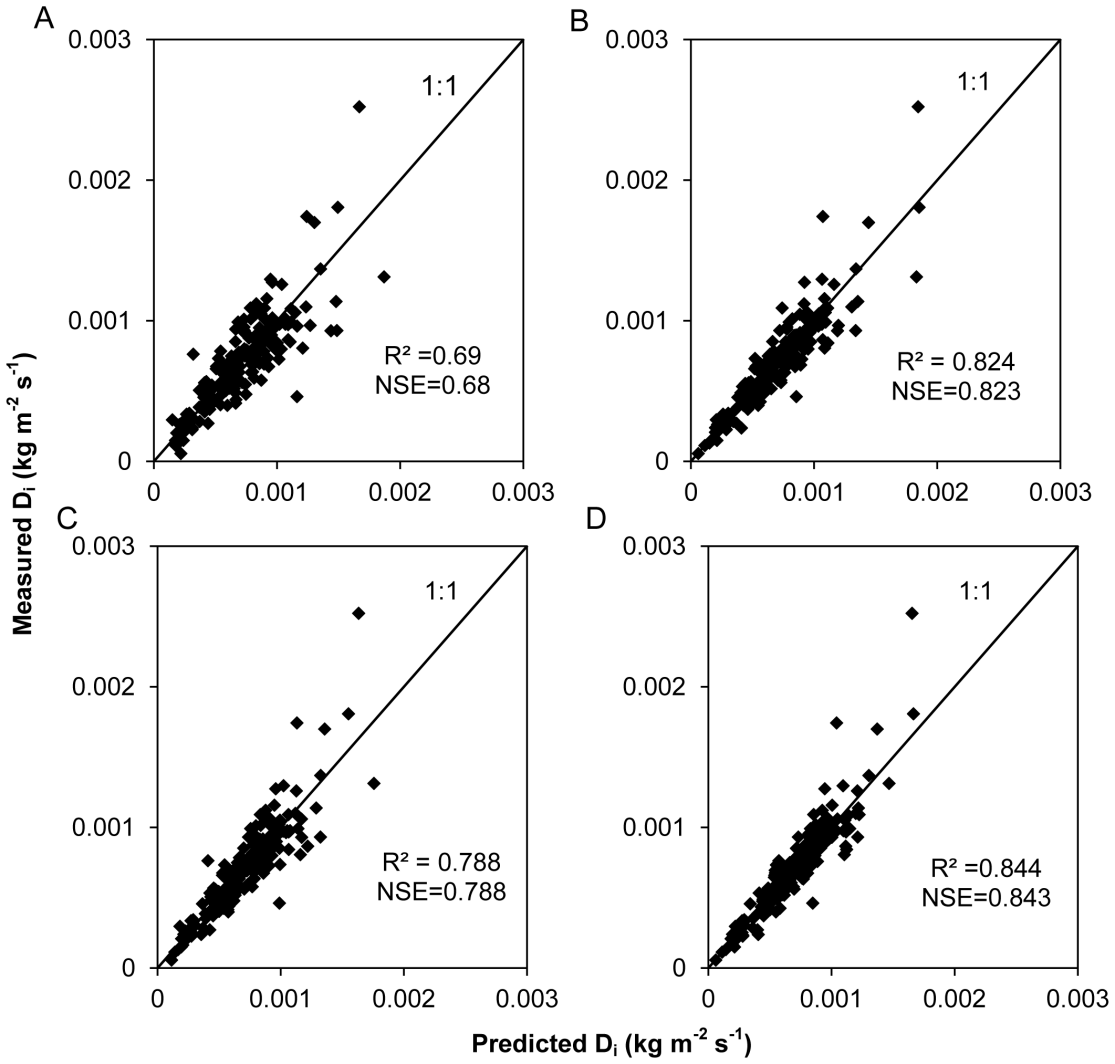
Measured vs. predicted interrill erosion rates. Measured and predicted interrill erosion rates for Eq. (1) (A), Eq. (3) (B), Eq. (4) (C), and Eq. (10) (D) using experimental data from WEPP cropland soil field interrill erodibility experiments.

The “intrinsic” interrill erodibility for a given soil is independent of runoff rate, rainfall intensity, infiltration, slope length, and gradient. If an interrill erosion equation adequately describes the relationship between interrill erosion rate and its influencing factors, the interrill erodibility, as estimated by an interrill erosion equation for a given soil, should change little over a wide range of conditions. Therefore, the CV of interrill erodibility estimated from an interrill erosion equation for a given soil under a wide range of conditions can be used to judge the quality of the interrill erosion equation. Interrill erodibility values, as estimated from the different interrill erosion equations discussed in this paper, along with their CV for 33 soils in the United States, are given in Table 6. In general, there were common trends in the interrill erodibilities predicted by the various interrill erosion equations. For all soils, very strong positive correlations (p<0.05) were found between the interrill erodibility values predicted from the various interrill erosion equations. Although the trends showed that interrill erodibility values predicted from the different interrill erosion equations reflected the strength of soil resistance to interrill erosion, the precision of the predictions was different among the equations. The CV of interrill erodibility from Eq. (10) was less than that from Eq. (1) for 30 soils, that from Eq. (10) was less than that from Eq. (3) for 25 soils, and that from Eq. (10) was less than that from Eq. (4) for 29 soils. For 23 soils, the CV of interrill erosion from Eq. (10) was the lowest among those from the four equations. Figure 8 shows the observed and predicted values for the various equations. The correlation coefficients and Nash-Sutcliffe coefficients between the measured and predicted values for Eq. (10) were the highest among all the equations, which suggested that Eq. (10) described the relationship between interrill erosion rate and its influencing factors better than previously proposed interrill erosion equations, even though the slope length of the plots in the experiments used for validation was fixed.

**Table 6:** Interrill erodibility and its CV from the different interrill erosion equations for the experimental data from the WEPP cropland soil field interrill erodibility experiments.

Soil type	No. of plots	*D*_i_ = *K*_i_ *I* ^2^ *S*_f_		*D*_i_ = *K*_i_ *RIS*_f_		*D*_i_ = *K*_i_ *q* ^1/2^ *IS* ^2/3^		*D*_i_ = *K*_i_ *RI* ^0.22^ *S*_f_ *L* ^−0.25^	
		K_i_	CV (%)	K_i_	CV (%)	K_i_	CV (%)	K_i_	CV (%)
Sharpsburg	6	1,917,372	24.05	2,457,363	13.22	23,850	17.18	323	13.03
Hersh	6	4,153,649	37.64	6,801,244	31.43	58,530	31.28	920	26.69
Keith	6	3,403,141	23.66	4,229,851	24.27	44,048	21.04	597	19.94
Amarillo	6	4,213,438	31.33	6,235,421	33.63	65,118	31.16	865	34.25
Woodward	6	4,592,143	27.85	7,568,362	20.60	74,617	28.95	1,049	27.73
Heiden	6	1,336,998	24.65	1,638,827	30.53	17,529	25.49	211	30.66
Whitney	6	3,101,189	20.41	4,367,166	16.02	48,903	18.38	590	14.56
Academy	6	2,994,017	25.59	3,932,019	11.12	40,099	16.97	514	9.04
Los Banos	6	2,405,840	14.00	3,262,543	17.95	30,191	10.92	414	10.58
Portneuf	6	1,382,542	16.90	2,314,773	14.54	20,975	13.61	280	12.42
Nansene	6	3,206,879	22.89	4,936,611	16.09	44,005	15.82	682	11.03
Palouse	6	4,003,363	18.95	5,201,600	12.97	48,338	13.65	682	9.57
Zahl	6	3,267,617	29.70	3,840,678	20.37	42,288	21.38	512	14.65
Pierre	6	2,300,664	17.06	2,719,824	7.11	27,829	11.27	367	6.60
Williams	6	2,684,238	24.36	3,548,762	11.90	38,403	10.74	508	10.17
Barnes - ND	6	1,876,045	10.85	3,056,669	10.09	31,502	8.41	474	8.12
Sverdrup	6	2,362,425	17.69	3,958,183	14.65	34,180	13.69	533	14.54
Barnes - MN	6	1,503,947	21.63	2,576,945	20.84	24,530	18.80	409	17.40
Mexico	6	2,970,456	16.24	4,153,863	15.04	41,848	13.23	570	12.10
Grenada	6	2,633,592	19.66	3,372,939	10.67	35,349	13.24	462	10.24
Tifton	6	652,793	54.22	1,603,183	27.15	12,053	36.29	221	23.88
Bonifay	6	871,497	73.08	4,226,263	15.32	22,179	47.29	581	21.05
Cecil	6	1,858,964	25.03	2,282,079	18.68	24,807	21.10	319	19.42
Hiwassee	6	1,873,194	18.16	2,243,785	17.27	25,030	15.54	320	13.94
Gaston	6	2,036,993	13.15	2,205,358	16.98	24,961	14.49	297	16.56
Opequon	6	3,195,673	16.05	3,398,535	9.63	39,643	11.97	474	7.20
Frederick	6	2,477,747	29.91	3,331,759	15.01	35,043	21.67	476	14.22
Manor	6	2,694,182	23.21	3,563,361	12.63	38,111	17.67	515	13.31
Caribou	6	1,551,246	11.13	1,845,618	7.78	20,635	8.21	263	7.82
Collamer	6	3,456,598	16.26	4,121,058	9.61	44,942	10.17	566	6.60
Miamian	6	1,591,767	22.99	2284814	18.64	22548	20.61	310	19.44
Lewisburg	6	2,257,824	20.43	2,803,097	15.93	29,807	16.97	381	14.62
Miami	6	1,970,749	26.84	2,579,164	18.97	26,927	20.07	356	15.01

The CV of interrill erodibility from Eqs. (3) and (10) was generally less than that from Eq. (1), indicating that rainfall intensity or flow discharge alone cannot completely express interrill erosion processes. Compared with Eq. (3), the reduced proportion of flow discharge in Eq. (4) resulted in an increase in the CV of interrill erodibility, but the reduced proportion of rainfall intensity in Eq. (10) resulted in a decrease in the CV of interrill erodibility. These results indicated that flow discharge is still the most important transport agent for interrill erosion processes, even though rainfall intensity has two effects, i.e., detachment of soil and enhancement of the transport capacity of thin overland flow. The previously proposed interrill erosion equations overemphasized the effect of rainfall intensity on interrill erosion when slope length was relatively longer. The CVs of interrill erodibility from Eq. (3) and Eq. (10) were closer, perhaps because slope length was fixed in the WEPP cropland soil field interrill erodibility experiments.

### Conclusions

Previously proposed interrill erosion equations based on mini-plot experiments largely ignored the effect of slope length and plot size on interrill erosion rate. A series of simulated rainfall experiments was conducted to validate the previously proposed interrill erosion equations. The results indicated that slope gradient almost adequately predicted interrill erosion in the previously proposed equations, but that the interrill erodibilities calculated from previously proposed equations generally decreased with increasing slope length and rainfall intensity. This result suggested that adding a factor for slope length and adjusting the factor for rainfall intensity are necessary to improve prediction of interrill erosion rate. An improved interrill erosion equation, 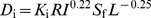, was developed by analyzing the relationships between sediment concentration and rainfall intensity, slope length, and slope gradient. To evaluate the effectiveness of the improved interrill erosion equation, part experimental data from WEPP cropland soil field interrill erodibility experiments were used for validation. The results indicated that the improved interrill erosion equation predicted interrill erosion rate better than previously proposed interrill erosion equations, even though the slope length of the plots in the WEPP experiments was fixed.
